# Bilberry/red grape juice decreases plasma biomarkers of inflammation and tissue damage in aged men with subjective memory impairment –a randomized clinical trial

**DOI:** 10.1186/s40795-021-00482-8

**Published:** 2021-11-22

**Authors:** Siv K. Bøhn, Mari C. W. Myhrstad, Magne Thoresen, Iris Erlund, Ann Kristin Vasstrand, Anne Marciuch, Monica H. Carlsen, Nasser E. Bastani, Knut Engedal, Kjell M. Flekkøy, Rune Blomhoff

**Affiliations:** 1 Department of Chemistry, Biotechnology and Food Sciences, Norwegian University of Life Sciences, Ås, Norway; 2 Department of Nutrition, Faculty of Health Sciences, Oslo Metropolitan University, Oslo, Norway; 3 Department of Biostatistics, Institute of Basic Medical Sciences, Oslo, Norway; 4 Department of Government Services, Finnish Institute for Health and Welfare, Helsinki, Finland; 5 Department of Psychology, University of Oslo, Oslo, Norway; 6 Department of Nutrition, Institute of Basic Medical Sciences, University of Oslo, Oslo, Norway; 7 Department of geriatric medicine, Oslo university hospital, Oslo, Norway

**Keywords:** Bilberry; blueberry; blueberries, Grape, Cytokines, Memory, Inflammation, Aged men

## Abstract

**Background:**

Few randomized clinical trials have explored the health effects of bilberries in humans. The aim was to test the effect of bilberry and red grape-juice consumption on visual memory, motor speed and dexterity as well as inflammatory and tissue damage biomarkers of plasma in aged men with subjective memory impairment.

**Methods:**

Nine-week double-blind, placebo-controlled, dietary intervention study of aged men (*n* = 60, age ≥ 67 years) with subjective memory impairment randomized to consume a 50/50 mix of bilberry/red grape-juice or an iso-caloric placebo juice. A selection of Cambridge Cognition Test Battery (CANTAB), Grooved Pegboard tests and blood-sampling for biomarker analysis were performed before and after the intervention.

**Results:**

Compared to placebo the selected memory and motor test scores were un-affected by the bilberry/red grape intervention. However, the plasma levels of tissue damage biomarkers decreased significantly more in the bilberry/red grape group. In particular lactate dehydrogenase (LDH) decreased from 362 U/L (median, baseline) to 346 U/L (median, post intervention) in the bilberry/red grape group. Also, several biomarkers of inflammation (EGF, IL6, IL9, IL10 and TNFα) decreased significantly more in the bilberry/red grape group. Furthermore, several plasma polyphenols; p-coumaric acid, hippuric acid, protocatechuic acid, 3HPAA and vanillic acid, increased significantly more in the bilberry/red grape group compared to placebo with the largest increase in p-coumaric acid with 116%; from 2.2 [1.0,5.5] to 4.7 [2.8,8.1] μM/L (median [95% CL]).

**Conclusions:**

The results indicate that a nine-week bilberry/red grape juice intervention has no measurable effects on the selected memory scores in aged men experiencing memory problems but decreases the level of biomarkers of inflammation and tissue damage. Whether the dampening effects on inflammation and tissue damage biomarkers have relevance for neuroinflammatory brain pathology remains to be established.

**Trial registration:**

Registration number (ClinicalTrials.gov: NCT00972972), September 9, 2009.

**Supplementary Information:**

The online version contains supplementary material available at 10.1186/s40795-021-00482-8.

## Background

Dementia develops over decades through pre-dementia stages such as subjective cognitive impairment (SCI) and mild cognitive impairment (MCI) [1–3] which can either be reversed, haltered or deteriorate further into Alzheimer’s disease (AD) [4]. In Norway the standardized prevalence of dementia and MCI in those above 70 years of age was recently reported to be 14.6 and 35.3%, respectively [5]. Although AD research is rapidly progressing [6] it remains unclear what are the major causes of the disease. However, the pathology of AD is associated with increased oxidative damage and inflammation [7].

In epidemiological studies plant-rich diets have consistently been associated with lower risk of oxidative stress and inflammation associated diseases, such as AD [8] and the therapeutic potential for neuroprotective effects of natural compounds and plant-food has been studied in several clinical trials [9, 10]. Particularly flavonoid-rich food, has been associated with a dose-dependent higher performance on several cognitive tests [11]. Animal studies have shown that intake of plant food (e.g., blueberries, strawberries and spinach) can retard and even reverse age-related decline in brain function and cognitive and motor performance in rats [12]. Recently a compound of pomegranate was found to increase the process of eliminating damaged mitochondria in the hippocampus of AD mice and improve learning and memory [13].

Two candidate polyphenol-rich food items, European wild blueberries (*Vaccinium Myrtillus*) (i.e bilberries) and grapes, have been particularly promising in aspect of neuroprotective effects in animals [14, 15]. Resveratrol, a phenolic compound enriched in the skin of grapes, prevents or slows the progression of a number of age-related diseases across animal species and disease models [16, 17]. Clinical trials with grape products [18, 19] and bilberry products [20, 21] also suggest that these plant foods may beneficially modulate oxidative damage and dampen inflammation. However while polyphenol-rich food-items such as blueberries/bilberries and grapes and their constituents have potential for impacting many aspects of health [18–26], further studies are called upon to gather a better understanding of the real impact following ingestion [27].

Clinical trials that tests effects on cognitive measures typically use standardized tools to test visuospatial learning and episodic memory such as CANTAB [28] sometimes combined with measures of verbal learning and spatial memory. A few small human clinical trials have been conducted to assess the effects of grape juice [29, 30] and bilberry juice [31] on cognitive measures. Both grape- and bilberry-juice consumption improved verbal learning in aged volunteers with mild cognitive memory impairment [29, 31] and grape-juice intake also improved spatial memory in a 12 week crossover trial of 25 middle aged women [30]. Recently a double blinded, placebo-controlled 6 months trial with wild blueberry and grape extracts in healthy elderly found improvements in some measures of memory particularly in those with the lowest memory performance [32].

To our knowledge the combined effects of blueberry and grapes juice on cognitive measures and biomarkers of oxidative stress and inflammation has not been tested in a human clinical trial before. We have therefore performed a double-blinded placebo-controlled intervention in a population of aged men with subjective memory impairment (SMI), i.e. symptoms of subjective cognitive impairment (SCI) to test the hypothesis that polyphenol-rich juice made from European wild blueberries, (*Vaccinium Myrtillus*) and red grape *(Vitis Vinifera)* beneficially affects memory, fine motor skills and reduces biomarkers of oxidative stress, inflammation and tissue damage.

## Methods

### Population

Aged men with SMI were recruited via advertisement in two national newspapers, in December 2006, and interviewed on telephone prior to entering the study regarding type of work, years of education, injuries and disease incidences and mental status using a modified version of the Folstein Mini-Mental State Examination (MMSE) [33]. Inclusion criteria were men of Norwegian ethnicity between 67 and 77 years of age with SMI and living in the Oslo-area (Oslo, Norway). The exclusion criteria were prior head-trauma with loss of consciousness, brain stroke within the last 3 years, symptoms of dementia or other brain-degenerative illnesses, cognitive deficit as defined by MMSE score below 26, depression as defined by Montgomery Asberg Depression Rating Scale (MADRS) of 20 and above, reported continuing treatment for cancer (cytostatica), diabetes, major psychiatric illness, alcohol/narcotic drug abuse, motor or sensory handicaps of relevance for testing, or blueberry or grape used as dietary supplements. Subjects with allergy to the intervention items were also excluded, as were those who reported extreme dietary habits as part of their lifestyle.

### Design

**Fig. 1:**
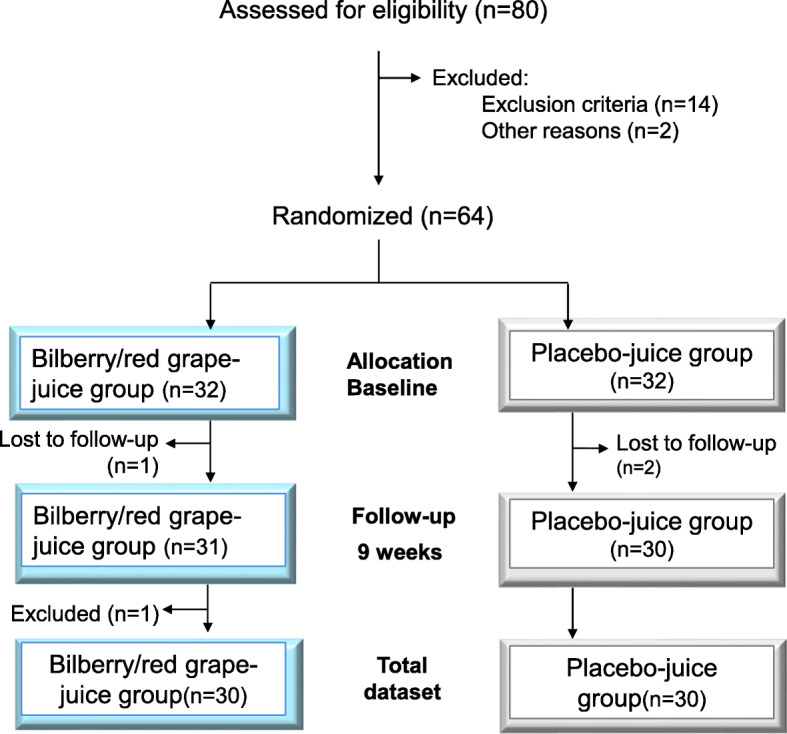
Overview of the study design

This 9 week double blinded placebo controlled intervention study followed a randomized parallel group design (Fig.1) and was performed during the time period December 2006 – July 2007. The study adheres to CONSORT guidelines (www.consort-statement.org).

Out of 80 participants assessed for eligibility over a telephone interview, 14 were excluded due to exclusion criteria leaving 66 individuals to be invited to an introductory meeting at the study center. Sixty-four individuals showed up for the introductory meeting which included information about the study and completing the informed consent. Then a semi-structured interview was performed to register demographic data, former and present diseases and level of self-reported memory problems. MMSE and MADRS analysis were performed for a final assessment with regards to the exclusion criteria.

As part of the introductory meeting a pretest screening was performed for assessment of eligibility. The subjects were asked to evaluate their memory problems at present and the degree to which memory functions had changed as compared to earlier years. In neuropsychological terms, these memory problems would be described as moderate. Furthermore, the neurological test battery was demonstrated for the participants serving as a simple introduction to the touch screen method in order to avoid bias of an initial learning curve between the baseline measurement and the re-test after end of the intervention. The introduction test also screened for visual, movement and comprehension difficulties which also served as exclusion criteria. The introductory meeting revealed that there was no further need for exclusion, leaving 64 participants to be randomly assigned to an intervention or a placebo group (32 subjects in each). The participants were given incremental unique ID-numbers from 1 to 64 based upon their scheduled appointment times. The participants of each group were tested at baseline, the day before entering the intervention part of the study and after 9 weeks (see Fig. 1). During the following week after baseline tests and sampling the participants received either juice boxes labelled “1” or juice boxes labelled “2” for 9 weeks consumption at their homes according to a pre-defined randomization list. Both juice types were packed in similar boxes, blinding the participants to the juice types. The information about the intervention status of the subjects was withheld from the experimenters until after all tests and analyses were performed.

### The intervention

The intervention group consumed 330 ml bilberry *(Vaccinium Myrtillus, European blueberries)* and red grape *(Vitis Vinifera, Bobal*, La Mancha, Spain*)*-juice twice a day with a total daily volume of 660 ml. The control group received the equal amount of placebo juice. The bilberry and red grape juice was composed of pure bilberry juice and red grape juice in equal amounts (with no added sugar or other additives) based on the scientific evidence demonstrating promising neuroprotective effects of both juice types in animal studies and also due to a potential better compliance when balancing the bitter taste of the bilberry juice with that of the grape juice. Both bilberry/red grape juice and placebo beverage were provided for this study by Tine BA, the largest food company in Norway (www.tine.no/english). Tine BA bought the raw material and produced the experimental juices based on the requirements from the leading investigators. The placebo beverage was formulated to look and taste like bilberry/red grape juice and to contain the same amount of carbohydrates and total energy. The placebo beverage contained 6.25 g sucrose, 6.25 g maltodextrine, 1.3 g citric acid (pH 3.0), 2.5 g Carmine solution E120 (4% carmine colouring agent), 0.025 g blueberry aroma, Potassium sorbate E202 and water. The amount of energy (50 kcal/100 g) and carbohydrates (12.5 g/100 g) were about the same as for the bilberry-grape juice. Both beverages were pasteurized at 95 °C for 14–15 s using Ultra-high temperature processing (UHT) technology and transferred to similar neutral containers (Tetra Prisma® Aseptic) under aseptic conditions. The containers were labeled either 1 or 2 before transportation to the homes of the participants. The juices were stored at cool temperatures for 9 weeks at the homes of the participants. The participants were asked to deliver a weekly report on juice consumption and leftovers together with comments on eventual side effects of the intervention drinks or other relevant problems. Participants were instructed not to consume bilberries or grapes during the introductory period of test training and prior to the baseline testing. No other restrictions on polyphenol intake were given during the study. Every second week the participants were contacted by telephone for motivation and follow-up. Sixty-one participants completed the study. Data from one participant in the bilberry/red grape group was excluded before data analysis due to intake of high-dose antiinflammatory agents.

### Registration of dietary habits

A food frequency questionnaire (FFQ) [34] was used to register the baseline dietary habits of the participants prior to the randomization of the groups. The form also asks for current diseases.

### Neuropsychological assessment

For assessment of memory functions a selection of computerized tests from the Cambridge Cognition Test Battery (CANTAB) were applied (www.camcog.com) using touch-sensitive computer screens. CANTAB is a validated, standardized PC-based test battery for assessment of verbal, visual and working memory. The battery has proven to be stable across testing sessions, avoiding ceiling- and floor-effects, and to be graded for intelligence [28]. Subtests have been found to be sensitive to neurodegenerative disorders like AD [35]. A computerized battery was chosen in order to standardize the testing condition across subjects. Tests for visual memory were selected and the numbers kept to a minimum to avoid fatigue and secure collaboration and motivation across the test-retest time span. For this reason, only visual memory was assessed. About a week prior to first testing, a short Motor Screening Test was administered to familiarize the subjects with the PC-format and testing procedures, reduce individual differences in adeptness for testing, and screen for visual, movement and comprehension difficulties. CANTAB tests and retests were administered in parallel versions to minimize test-retest learning effects. Instructions were presented verbally in Norwegian after having been translated from English to Norwegian and back to secure the same meaning in the two languages. The tests selected were presented as below:

1. **Delayed matching to sample** (DMS) measures forced-choice recognition memory of non-verbally coded visual patterns in a simultaneous and a delayed choice situation after 0, 4 and 12 s.
2. **Paired associates learing** (PAL) tests episodic memory and associative learning as well as the progression of learning of spatial localization and identity of visual patterns.
3. **Pattern recognition memory** (PRM) is a test for visual pattern recognition in a two-choice paradigm.
4. **Spatial recognition memory** (SRM) requires the subject to remember the spatial location of simple visual figures as a forced choice between two localities.
5. **Grooved Pegboard Test**, dominant and nondominant hand, from the Halstead-Reitan Neuropsychological Test Battery [36–38] was used to measure fine-motor speed and control.

### Sample collection and preparation

Over-night fasting blood samples were taken between 0730 and 0930 by a biomedical laboratory scientist at the accredited (NS-EN ISO/IEC 17025) Dr. V. Fürst Medical Laboratory, Oslo, Norway. Blood samples were collected at the time of randomization and at the end of the intervention period. BD Vacutainer® tubes were used to collect ethylenediaminetetraacetic acid (EDTA) samples (no. 368856), serum samples (no 367953), heparin samples (no 367869), EDTA samples for homocysteine analysis (no 362795), and samples for white blood cell count (no. 368856). The tubes were gently flipped 8–10 times before analysis. The same vacutainers were used to obtain EDTA plasma. Plasma and serum were collected, separated into aliquots, and immediately stored in 2 ml plastic tubes at − 70 °C until time of analysis.

### Measurement of plasma and serum biomarkers

The 8-epi-prostaglandin F2 (8-epi PGF_2α)_ isoprostanes concentrations were determined in EDTA plasma as previously described [39]. The enzymes aspartate aminotransferase (ASAT) /(AST/GOT), lactate dehydrogenase (LDH) and myocardium specific creatine kinase (CK-MB) were detected in heparin plasma using the MAXMAT PL compact platform system for Clinical Chemistry testing (www.maxmat.fr). All reagents and controls were purchased from MAXMAT. Analyses were performed according to instructions by the manufacturer. All controls were measured within the expected variation ranges. Total creatine kinase, CK _(total)_ was measured in serum by Fürst laboratory, as part of the ‘standard clinical parameters’ by a photometric kinetic UV-test using Roche Modular.

CK _(total)_ Homocysteine, High-density lipoprotein (HDL-C), Low-density lipoprotein (LDL-C), Total Cholesterol, γ-glutamyl transferase (γGT), Uric acid, Sodium, Potassium, Albumin, fasting Glucose, Micro-CRP and Creatinine were measured in serum while white blood cell count was performed on EDTA blood. Analyses were performed by the accredited (NS-EN ISO/IEC 17025) Dr. V. Fürst Medical Laboratory**,** Oslo, Norway. Carotenoid analysis were performed in heparin as previously described [40] and were performed by Vitas. EDTA plasma was used for polyphenol analysis. Quercetin was analyzed by high performance liquid chromatography (HPLC) and electrochemical detection after enzymatic hydrolysis as described elsewhere [41]. Phenolic acids and enterolactone were analyzed by gas chromatography-mass spectrometry after enzymatic hydrolysis using a modification of a previously described method [42]. Paraxanthine which is a methylxanthine was analyzed by HPLC [43].

The 21 cytokines were detected in heparine plasma using a sandwich immunoassay-based protein array system (Human Cytokine/Chemokine Multiplex Immunoassay Kit, MILLIPLEX MAP Human Cytokine/Chemokine Panel (Cat# MPXHCYTO-60 K, Milllipore, Billerica, USA) according to the manufacturer’s instruction. The Bio-Plex 100 System with the BIORAD manager acquisition program (BIORAD manager 4.1) was used to run the samples and process the data. Controls, standards and the samples were run in duplicates. The samples were randomized to three plexes with regard to intervention groups in order to avoid bias of intra-plex variations. Five participants were randomly excluded due to limitations in number of kits available. Pre- and post-intervention samples from each individual were run on the same plex.

### Sample size

At the time of study design there were no other existing studies measuring effects of diet intervention on memory scores in humans. Thus we based the sample size on a similar study performed in a population at risk of cardiovascular disease, investigating effects of 330 ml bilberry juice intake each day over a six-week period on biomarkers of inflammation [20]. In this study a reduction of 30% in IL-6 levels (− 6.0 pg/ml) was found in the bilberry group and a standard deviation for the change could be calculated to 8.55 pg/ml based on the range rule. Power calculation was performed using an online sample size calculator (http://powerandsamplesize.com/), using the calculation for 2 Means, 2-Sample, 2-Sided Equality. Based on these values, assuming no change in the placebo group, type I error α = 0.05 and a type II error β = 0.2 (80% power). Estimated sample size based on two-sided equality gave 32 in each group indicating that a total inclusion of 65 individuals, as managed in the current study, would be sufficient.

### Data analysis

Categorical variables are presented as frequencies with percentages. Continuous variables are presented as medians with 95%CL and the Mann-Whitney test was applied to compare the two groups. Results were considered significant with *p*-values below 0.05 and presented with three decimals. Chi-Square-tests and Fisher’s exact test were performed for categorical variables. All statistics were performed using the IBM SPSS software for Windows (version 26.0). Box plots were created using R version 3.4.4 with the package ggplot2.

## Results

### Study population

After assessing the responding participants (*n* = 80) for eligibility, 16 participants were excluded leaving 64 to be randomized into two groups of equal sizes. The study was conducted during the time period December 2006 – July 2007. Three participants were lost to follow-up and one was excluded from the dataset leaving a resulting dataset comprising 30 individuals in each group. The groups were comparable with regards to age, years of education, years of working, BMI and smoking status (Table 1). The study population was highly educated, with 17 and 16.5 years of education (median) in the intervention group and control group. In Norway, a person with 17 years of education has 3 years of upper secondary education and five years of higher education on top of the 9 years of compulsory primary and lower secondary school. The participants had median BMI within normal range and relatively few smokers. The participants in both groups had a median daily intake of more than 500 g of fruits and vegetables which is above the recommended intake by the Norwegian dietary guidelines.

**Table 1:** Population description

	Bilberry-grape juice (***n*** = 30)		Placebo juice (***n*** = 30)	
**Basic charachteristics**				
Age (years)	72	(69.0–75.0)	71	(69.0–73.0)
BMI	24	(23.8–26.0)	25	(24.5–26.5)
Education (years)	17	(16.0–18.0)	16.5	(15.0–17.0)
Work (years)	43	(40.0–46.0)	45	(44.0–49.0)
Memory problems (years)	2	(2.0–3.0)	3	(3.0–7.0)
**Smoking status**				
Non-smokers, (n)	26	(26%)	29	(29%)
Smokers, years (n)	4	(4%)	0	(0%)
NN(n)	0	(0%)	1	(1%)
**Former smoking status**				
Never smokers (n)	13	(13%)	11	(11%)
Former smokers (n)	15	(15%)	14	(14%)
**Smoking duration**				
Former smoking, (years)	22	(14.0–30.0)	20	(17.0–25.0)
Years since quitting smoking	35	(30.0–38.0)	31	(24.0–35.0)
**Comorbidities**				
Asthma (n)	0	(0%)	5	(17%)
Other airway diseases (n)	0	(0%)	2	(7%)
Stomach-related diseases (n)	1	(3%)	2	(7%)
Rheumatism (n)	3	(10%)	3	(10%)
Heart related diseases (n)	3	(10%)	3	(10%)
Stroke	0	(0%)	2	(7%)
Diabetes type 1 (n)	0	(0%)	2	(7%)
Diabetes type 2 (n)	1	(3%)	2	(7%)
Cancer (n)	4	(13%)	5	(17%)
**Dietary intake**				
FRAP (total antioxidant intake)	22	(20–26)	23	(19–28)
Potoatoes (g/day)	136	(98–173)	127	(112–172)
Vegetables (g/day)	215	(146–281)	150	(121–288)
’Fruit and berries (g/day)	338	(229–422)	393	(260–536)
Bilberries (g/day)	9	(4–21)	5	(2–10)
Grapes(g/day)	10	(6–16)	10	(5–29)
Fish (g/day)	95	(61–124)	84	(73–115)
Coffe, filter/instant (cups/day)	2	(2–4)	4	(4–6)
Meat (g/day)	102	(71–120)	110	(94–135)

Categorical variables are presented as frequencies with percentages. Continuous variables are presented as medians with 95%CL

### Baseline memory functions related to test-norms

At baseline, the total experimental population performed below the CANTAB age-adjusted test norms for DMS forced-choice, recognition memory all delays, and for Total correct after 4 s. (*p* < 0.01; data not shown). PAL First trial memory score, Total errors and Total errors for 6 shapes were also reduced (*p* < 0.05; data not shown).

### Intervention effects

#### Memory scores

No significant differences were found between the bilberry/red grape group and controls with regards to the changes in CANTAB memory scores (DMS, PAL, PRM and SRM) or in the results of the Grooved Pegboard tests (Table 2). The groups performed similarly at baseline.

**Table 2:** Neuropsychological test scores

	Bilberry/red grape juice (***n*** = 30)				Placebo juice (***n*** = 30)				
	Baseline		Post intervention		Baseline		Post intervention		p^**MW**^^(^^**change)**^
**CANTAB**									
***DMS***									
Total correct (all delays)	12.0	(12.0–13.0)	12.0	(11.0–13.0)	12	(12.0–13.0)	12.5	(11.0–13.0)	0.71
Total correct (4000 ms delay)	4.0	(4.0–5.0)	4.0	(4.0–5.0)	4.0	(4.0–5.0)	4.0	(4.0–5.0)	0.76
Mean correct latency (all delays)	3938	(3086–4367)	3486	(3193–4465)	4225	(3729–4715)	4134	(3692–4469)	0.29
***PAL***									
First trial memory score (n out of 22)	11.5	(10.0–13.0)	10.0	(10.0–13.0)	11.0	(10.0–14.0)	11.0	(10.0–14.0)	0.24
Mean trials to success (n out of 10)	2.6	(2.4–3.2)	3.0	(2.8–4.0)	2.4	(2.0–2.8)	2.4	(2.2–2.8)	0.16
Total errors (n adjusted)	25.0	(18.0–34.0)	31.5	(22.0–40.0)	19.5	(13.0–25.0)	23.5	(18.0–32.0)	0.36
Total errors (6 shapes, n adjusted)^a^	8.0	(7.0–11.0)	6.0	(4.0–9.0)	5.5	(4.0–9.0)	6.0	(6.0–11.0)	0.15
Total errors (8 shapes, n adjusted) ^b^	13.5	(9.0–20.0)	21.5	(16.0–29.0)	11.0	(9.0–16.0)	16.0	(15.0–24.0)	0.50
***PRM***									
Number correct (n)	22.0	(21.0–23.0)	21.5	(21.0–23.0)	21.0	(21.0–22.0)	21.0	(20.0–23.0)	0.47
Mean correct latency (ms)	2358	(2068–2609)	2305	(2090–2834)	2573	(2365–2708)	2302	(2050–2667)	0.15
***SRM***									
Number correct (n)	14.0	(14.0–16.0)	14.5	(13.0–16.0)	16.0	(16.0–18.0)	16.0	(16.0–18.0)	0.77
Mean correct latency	2538	(2266–2833)	2498.8	(2156–2855)	2416	(2234–2770)	2313	(1963–2504)	0.17
**PEGS**									
dominant hand (ms to accomplish test)	120.0	(95.4–140.4)	104.2	(89.9–126.0)	117.1	(98.2–127.2)	116.2	(96.5–126.1)	0.41
nondominant hand (ms to accomplish test)	124.2	(93.3–136.4)	116.6	(93.8–136.2)	113.4	(100.2–133.7)	107.2	(95.9–127.5)	0.52

Median baseline and post-intervention are given with corresponding 95%CL (lower, upper) separately for each group. **p**^**MW(change)**^ was obtained comparing the groups with regards to change using Mann Whitney test. No significant differences were found between the groups. DMS total correct = n of correct responses. Mean correct latency = ms to response. *CANTAB* Cambridge Cognition Test Battery, *DMS* Delayed Matching to Sample, *PAL* Paired Associates Learning, *PRM* Pattern Recognition Memory and *SRM* Spatial Recognition Memory, *PEGS* Grooved Pegboard Test. Adjusted; corrected for stages that were not accomplished. ^a^ all tests wrong result = max score of 60, ^b^all tests wrong result = max score of 80

#### Standard clinical parameters

The number of monocytes increased significantly in the bilberry/red grape group as compared to the control group (Table 3 and Supplemental Fig. 1). None of the other clinical parameters were statistically significantly changed between the groups during the intervention.

**Table 3:** Standard clinical biomarkers and cell counts

	Bilberry/red grape juice (***n*** = 30)				Placebo-juice(***n*** = 30)				
	Baseline		Post-intervention		Baseline		Post-intervention		p^MW(change)^
**Standard clinical biomarkers**									
Potassium (mM)	4.3	(4.2–4.5)	4.4	(4.2–4.5)	4.3	(4.2–4.6)	4.4	(4.4–4.6)	0.42
Sodium (mM)	142.0	(142.0–144.0)	142.5	(141.0–144.0)	142.0	(142.0–144.0)	143.0	(143.0–145.0)	0.23
fGlucose (mM)	5.2	(5.0–5.5)	5.1	(5.0–5.5)	5.4	(4.8–6.0)	5.1	(4.8–5.4)	0.74
Insulin (mU/L)	5.6	(5.0–6.7)	5.2	(4.2–5.9)	6.0	(3.9–8.4)	6.2	(3.9–7.6)	0.51
Cholesterol (mM)	5.9	(5.3–6.6)	5.6	(5.1–6.2)	5.5	(5.0–6.1)	5.5	(4.9–5.8)	0.22
S-Creatinin (mM)	85.0	(83.0–94.0)	86.5	(80.0–93.0)	90.5	(85.0–95.0)	90.0	(88.0–95.0)	0.97
fS-Triglycerids	1.0	(0.8–1.2)	0.9	(0.9–1.2)	0.9	(0.8–1.12)	1.0	(0.8–1.2)	0.49
S-Uric acid (mM)	338.0	(302.0–362.0)	346.0	(337.0–374.0)	346.5	(318.0–370.0)	371.0	(344.0–390.0)	0.60
S-Albumin (g/L)	42.0	(42.0–43.0)	41.0	(41.0–42.0)	42.0	(41.0–43.0)	41.0	(40.0–42.0)	0.38
S-HDL cholesterol (mM)	1.7	(1.6–1.9)	1.6	(1.6–2.0)	1.6	(1.4–2.0)	1.6	(1.4–1.8)	0.91
P-Homocystein (mM)	12.1	(11.5–14.5)	13.8	(12.4–15.2)	13.9	(11.8–15.3)	14.8	(12.6–16.5)	0.89
S-MikroCRP (mgL)	1.4	(1.1–1.8)	1.4	(1.0–1.7)	1.4	(0.9–2.1)	1.3	(1.0–2.4)	0.88
**Cell count (109/L)**									
Leukocytes	5.4	(4.6–5.9)	5.4	(4.7–5.9)	4.8	(4.5–6.2)	4.9	(4.5–5.7)	0.30
Basofiles	0.0	(0.0–0.0)	0.0	(0.0–0.0)	0.0	(0.0–0.0)	0.0	(0.0–0.0)	0.20
Lymphocytes	1.7	(1.4–2.0)	1.8	(1.6–2.0)	1.6	(1.5–1.9)	1.7	(1.3–1.9)	0.62
Eosinophiles	0.2	(0.2–0.3)	0.2	(0.2–0.3)	0.2	(0.2–0.3)	0.2	(0.2–0.3)	0.22
Neutrophiles	2.6	(2.2–3.3)	2.9	(2.6–3.1)	2.8	(2.4–3.4)	2.7	(2.4–3.0)	0.11
Monocytes	0.5	(0.5–0.6)	0.5	(0.5–0.6)	0.5	(0.5–0.6)	0.5	(0.5–0.6)	0.03*

Median baseline and post-intervention values are given with corresponding 95%CL (Lower, upper). p^MW(change)^ was obtained comparing the groups with regards to change using Mann Whitney test.*p < 0.05

#### Carotenoids, tocopherols and polyphenols

No difference between the groups were found with regards to changes in biomarkers of fruit and vegetable intake (lycopene, zeaxanthin, cryptoxanthin and α- and β-carotene) (Table 4) while the levels of lutein decreased in the bilberry/red grape group as compared to the control group (Supplemental Fig. 2 A).

**Table 4:** Plasma dietary α-tocopherol, carotenoids and polyphenols

	Bilberry/red grape juice (***n*** = 30)				Placebo-juice (***n*** = 30)				
	Baseline		Post-intervention		Baseline		Post intervention		p^MW(Change)^
α-tocopherol (μM)	31.4	(29.0–35.6)	30.0	(27.0–32.4)	30.5	(27.1–32.2)	28.8	(25.7–31.2)	0.38
**Carotenoids**									
lutein (μM)	0.24	(0.21–0.30)	0.22	(0.19–0.30)	0.26	(0.22–0.28)	0.27	(0.20–0.31)	0.04*
zeaxanthin (μM)	0.06	(0.05–0.08)	0.61	(0.04–0.07)	0.06	(0.06–0.08)	0.06	(0.05–0.07)	0.52
β-cryp (μM)	0.30	(0.23–0.38)	0.16	(0.12–0.26)	0.26	(0.20–0.38)	0.13	(0.09–0.20)	0.57
α-caroten (μM)	0.09	(0.52–0.13)	0.07	(0.05–0.10)	0.08	(0.05–0.12)	0.08	(0.04–0.09)	0.94
β-caroten (μM)	0.49	(0.33–0.73)	0.37	(0.30–0.50)	0.39	(0.30–0.73)	0.35	(0.26–0.62)	0.80
lycopen (μM)	0.55	(0.43–0.68)	0.53	(0.39–0.65)	0.57	(0.43–0.66)	0.51	(0.35–0.58)	0.21
**Polyphenols**									
Quercetin (nmol/L)	10.2	(5.2–14.8)	18.8	(11.6–24.8)	13.7	(8.1.-26.1)	10.2	(7.1–20.1)	0.27
3HPAA (nmol/L)	133.0	(85.1–178.8)	219.0	(148.0–268.7)	173.7	(107.3–244.7)	118.1	(68.8–153.2)	< 0.01**
33HPPA (nmol/L)	257.1	(104.0–569.0)	166.6	(75.9–266.2)	230.0	(89.7–350.2)	198.5	(107.1–437.1)	0.07
Vanillic acid (nmol/L)	41.2	(32.9–47.5)	44.3	(34.8–66.2)	41.5	(31.4–65.8)	44.4	(30.7–52.8)	0.03*
Protocatechuic acid (nmol/L)	30.8	(26.4–33.1)	42.0	(33.5–48.7)	36.7	(31.23–45.71)	30.2	(26.4–38.36)	< 0.01**
HVA (nmol/L)	65.8	(60.4–80.0)	89.7	(63.0–104.5)	61.0	(54.4–92.3)	63.9	(55.9–88.2	0.08
DOPAC (nmol/L)	98.7	(87.8–118.0)	125.3	(99.0–138.6)	105.1	(94.7–132.5)	112.3	(107.5–134.7)	0.28
Gallic acid (nmol/L)	4.3	(3.4–6.3)	4.5	(3.3–5.3)	5.3	(3.7–6.7)	4.8	(2.9–6.2)	0.46
DHCA (nmol/L)	24.1	(16.9–45.0)	26.7	(16.7–43.4)	40.3	(17.6–51.8)	26.7	(21.8–48.0)	0.81
p-Coumaric acid (nmol/L)	2.2	(1.0–5.5)	4.7	(2.8–8.0)	2.7	(1.3–5.9)	3.5	(2.6–5.0)	< 0.01**
Caffeic acid (nmol/L)	33.5	(20.6–38.2)	33.9	(24.4–46.8)	34.6	(24.4–55.6)	29.1	(17.1–45.9)	0.10
Ferulic acid (nmol/L)	23.6	(20.3–29.1)	22.0	(17.4–27.8)	24.5	(17.0–45.6)	18.1	(14.9–25.9)	0.18
Enterolactone (nmol/L)	17.5	(13.6–26.3)	25.6	(17.45–39.1)	30.7	(20.8–50.1)	30.7	(17.2–41.7)	0.06
Hippuric acid (nmol/L)	1674	(1185–2186)	2503.7	(1650–4442)	1354	(943–2380)	1367	(855–2504)	0.05*
Paraxanthine (μmol/L)	3.0	(1.8–4.6)	2.9	(2.0–5.6)	3.8	(2.0–5.3)	4.2	(2.7–6.2)	0.14

Data are presented as baseline median or median to post-intervention with corresponding 95%CL (lower, upper). p^MW(change)^ was obtained comparing the groups with regards to change using Mann Whitney test. *33HPPA* 3-(3-hydroxyphenyl)-propionic acid, *3HPAA* 3-hydroxyphenylacetic acid, *HVA* Homovanillic acid/4-hydroxy-3-methoxyphenylacetic acid*, DOPAC* 3,4-dihydroxyphenylacetic acid, *DHCA* Dihydrocaffeic acid, *PA* Protocatechuic acid. **Paraxanthine is a methylxanthine. *p ≤ 0.05, **p < 0.001

When compared to the changes in the control group we find several plasma polyphenols to be significantly increased in the bilberry/red grape group (Table 4, Supplemental Fig. 2 B-F). Based on the change from median baseline to median follow-up, using the median baseline as reference (Table 4), the level of hippuric acid increased with 50%, vanillic acid increased with 8%, protocathechuic acid increased with 36%, 3HPAA increased with 65% and p-coumaric acid increased with 116%. The baseline levels for all parameters were comparable between the groups except for plasma protocatechuic acid which was 19% higher in the control group.

#### Tissue damage biomarkers

**Fig. 2:**
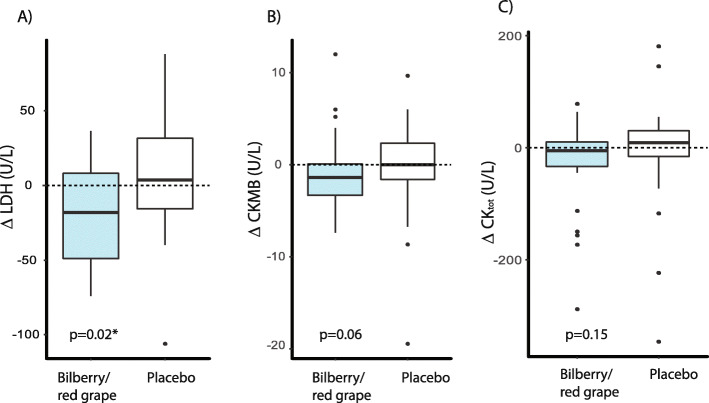
Change in plama tissue damage biomarkers;  lactate dehydrogenase (LDH) (**A**), myocard specific creatinine kinase  (CKMB) **(B**), total creatinine kinase  (CK total) (**C**) in the bilberry/red grape juice group (blue boxes) compared to the placebo group (white boxes). *P*-values are obtained by non-parametric MW tests

Baseline and post-intervention values for the tissue damage biomarkers are presented in Table 5 and the change were compared between the groups by MW test. The changes in tissue damage biomarkers (post-intervention – baseline) for each of the groups are presented as boxplots in Fig. 2. The tissue damage biomarker, LDH_,_ decreased significantly in the bilberry/red grape group when compared to the control group. The LDH levels decreased from 362 U/L (median baseline) to 346 U/L (median, post intervention) in the bilberry/red grape group. Compared to the placebo group there was also a strong trend for a higher decrease in the bilberry/red grape group for myocardium specific CK (CK-MB) (*p* = 0.055) decreasing from median 9.8 U/L at baseline (U/L) to 9.0 U/L (Table 5). Plasma creatine kinase (Ck_total_) also showed a trend to decrease more in the bilberry/red grape group (*p* = 0.151) (Fig. 2 C) while ASAT did not change differentially between the groups.

**Table 5:** Biomarkers of oxidative stress and tissue damage

	Bilberry-red grape juice (***n*** = 30)				Placebo-juice (***n*** = 30)				
	Baseline		Post intervention		Baseline		Post intervention		pMW^(Change)^
**Isoprostanes (ng/mL)**	94.3	(79.8–101.0)	83.6	(78.8–98.7)	86.4	(78.8–100.0)	82.6	(71.8–92.2)	0.54
**ASAT (U/L)**	24.5	(23.5–26.5)	23.5	(21.5–26.5)	25.8	(22.5–29.5)	25.3	(23.5–27.0)	0.49
**CKMB (U/L)**	9.8	(7.7–11.7)	9.0	(7.3–11.0)	10.0	(8.7–11.3)	10.5	(9.0–13.7)	0.06
**CK**_**tot**_ **(U/L)**	99.0	(84.0–134.0)	101.5	(83.0–137.0)	116.5	(90.0–147.0)	119.5	(111.0–167.0)	0.15
**LDH (U/L)**	361.8	(328.0–388.0)	345.8	(331.5–364.0)	334.5	(317.0–368.5)	346.5	(337.3–366.0)	0.02*
**γ GT (U/L)**	23.5	(20.0–28.0)	24.0	(22.0–34.0)	25.5	(19.0–39.0)	26.0	(22.0–43.0)	0.51

Data is presented as median for baseline and post intervention with the corresponding CL (Lower, upper). p^MW(change)^ was obtained comparing the change between the groups using the Mann Whitney test. *ASAT* Aspartate Aminotransferase, *CKMB* creatine kinase (myocard specific), *CK*_*tot*_ Creatine kinase total, *LDH* lactate dehydrogenase, *γ-GT*; gamma glutamyl transferase, *:*p* < 0.05

#### Correlation between memory functions and inflammatory biomarkers

Significant positive correlations between ‘PAL mean trials to success’ and/or ‘Pal total errors’ were observed for IL6, IL8, IL17, FGF2, GM-CSF, TNFα, IL9, IFNγ and VEGF (nonparametric correlation analysis, data not shown). This might indicate a possible association between higher inflammatory levels and impaired memory skills. IL9 and IL12 negatively correlated with ‘SRM number correct’ indicating lower memory skills with higher IL9 and IL12 levels (nonparametric correlation analysis, data not shown).

#### Cytokines

**Fig. 3:**
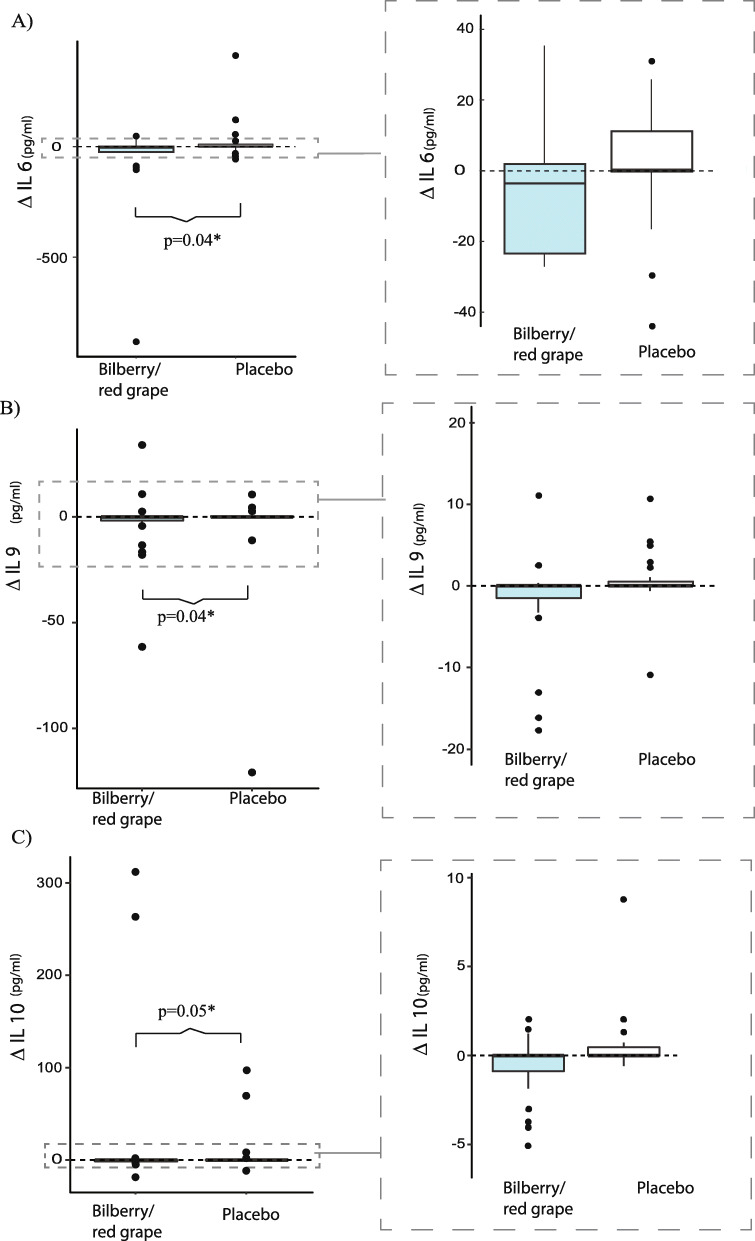
Change in plasma interleukins for: (**A**) Interleukin 6 (IL6), (**B**) IL9 and (**C**) IL10 in the bilberry/red grape juice group (blue boxes) compared to the placebo group (white boxes). *P*-values are obtained by non-parametric MW tests

**Fig. 4:**
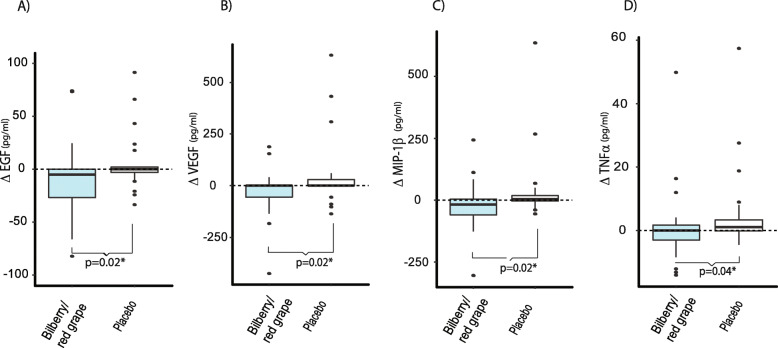
Change in plasma cytokins: (**A**) epidermal growth factor (EGF), (**B**) Vascular endothelial growth factor (VEGF), (**C**) Macrophage Inflammatory Protein (MIP-1β) and (D) tumor necrosis factor (TNFα)  in the bilberry/red grape juice group (blue boxes) compared to the placebo group (white boxes). P-values are obtained by non-parametric MW tests

Baseline and post-intervention median values are presented in Table 6 with their corresponding range. Several interleukins decreased in the bilberry/red grape group as compared to controls. The cytokines that were changed differentially between the groups are presented as boxplots based on the delta values (post-intervention – baseline) in Figs. 3 and 4. These were IL6, IL9 and IL10 (Fig. 3) and EGF, MIP1β, and TNFα (Fig. 4). Median IL6 was reduced from 8.1 pg/ml at baseline to 24.2 pg/ml post intervention in the bilberry group (Table 6). Median IL10 reduced from 1.1 pg/ml at baseline to 0.7 pg/ml post intervention and IL9 reduced from 1.2 to 0.6 pg/ml (Table 6). Note that while Table 6 indicates an increase in VEGF from baseline to post-intervention in the bilberry/red grape group looking at the group median, Fig. 4 shows that the median change in the two groups are the same and the significant difference between the groups is caused by a heavier tail towards decrease in the bilberry/red grape group and the opposite in the placebo group. Additionally, several plasma cytokines showed a trend (MW, *p* < 0.2) to decrease more in the bilberry/red grape group i.e. IL8, CD40L, IP10, IL12, IL17 and IFNγ.

**Table 6:** Cytokines

	Bilberry/red grape juice (***n*** = 29)				Placebo-juice (***n*** = 27)				
	Baseline		Post-intervention		Baseline		Post-intervention		p^MW(Change)^
**Growth factors (pg/ml)**									
EGF	40.5	(5.4–116.8)	23.6	(3.6–104.6)	10.6	(0.6–59.1)	8.5	(0.6–61.1)	0.02*
FGF2	29.7	(19.9–83.2)	33.0	(20.0–68.0)	21.3	(15.6–26.0)	20.1	(4.8–27.7)	0.26
GM-CSF	128.1	(40.6–706.3)	120.2	(44.1–268.4)	71.1	(32.9–129.7)	44.5	(22.3–118.3)	0.66
VEGF	92.4	(20.6–288.8)	124.6	(3.0–212.6)	114.5	(3.0–289.7)	93.3	(3.2–299.1)	0.02*
**Chemokines (pg/ml)**									
Eotaxin	902.6	(508.4–1999)	958.5	(466.9–2524)	656.7	(602.7–1025)	703.8	(541.7–1028)	0.29
Fractalkine	66.6	(3.6–405.7)	66.6	(3.6–405.7)	29.0	(3.6–344.2)	29.0	(3.6–344.2)	0.30
IL8	11.3	(6.6–28.2)	13.0	(8.1–24.3)	8.7	(6.5–13.7)	9.5	(7.8–16.9)	0.09
CD40ligand	488.5	(417.0–754.6)	513.3	(347.9–608.9)	508.1	(350.8–641.2)	491.4	(461.5–718.5)	0.09
IP10	1233	(1030–1848)	1118	(974–1442)	989	(833–1346)	1080	(997–1397)	0.13
MCP	409.2	(378.6–454.6)	398.4	(385.9–422.6)	415.4	(396.6–515.6)	450.8	(401.3–523.0)	0.55
MIP1b	110.6	(99.5–306.4)	131.1	(61.7–343.2)	72.1	(52.6–142.9)	93.7	(50.0–137.7)	0.02*
**Interleukins (pg/ml)**									
IL4	0.6	[0.6, 317.2]	0.6	[0.6, 353.5]	0.6	[0.6, 620.8]	0.6	[0.6, 210.3]	0.76
IL6	38.1	(4.0–164.2)	24.2	(5.4–170.4)	17.1	(5.2–44.6)	17.6	(4.0–49.9)	0.04*
IL7	3.7	(3.7–35.7)	3.7	(3.7–25.9)	0.6	(0.0–0.0)	0.6	(0.0–0.0)	0.17
IL9	1.2	(0.6–7.7)	0.6	(0.6–9.7)	0.6	(0.6–4.8)	0.6	(0.6–5.5)	0.05*
IL10	1.1	(0.7–4.9)	0.7	(0.7–1.9)	0.7	(0.7–3.3)	0.8	(0.7–3.1)	0.05*
IL12	13.6	(0.6–80.2)	7.4	(0.6–59.7)	1.9	(0.6–15.1)	1.8	(0.6–17.7)	0.15
IL13	2.4	(0.6–25.2)	0.6	(0.6–39.3)	0.6	(0.6–9.9)	0.6	(0.6–5.5)	0.30
IL17	11.2	(4.7–45.7)	13.5	(4.5–39.6)	10.8	(1.5–29.9)	11.7	(1.6–23.2)	0.17
TNF α	8.2	(5.1–24.8)	7.5	(6.1–24.8)	6.0	(5.0–9.0)	6.8	(4.5–9.4)	0.04*
IFN γ	17.6	(5.7–134.7)	15.7	(6.4–103.6)	9.0	(2.0–17.9)	9.7	(2.3–15.6)	0.06

Data is presented as median for baseline and post intervention with the corresponding 95%CL (lower, upper) or [min, max]. *P* values are given for the comparison of changes between the groups (Mann Whitney test). *EGF* epidermal growth factor, *FGF* fibroblast growth factor, *GM-CSF* Granulocyte-macrophage colony-stimulating factor, *VEGF* Vascular endothelial growth factor, *IP* immunoprotein, *MCP* monocyte chemotactic protein, *MIP* Macrophage Inflammatory Protein, *IL* interleukin, *TNF* tumor necrosis factor, *IFN* Interferon. *p ≤ 0.05

#### Compliance and tolerance

Out of all the participants that completed the study 77% (25 in the bilberry/red grape group and 22 in the control group) reported to drink the amounts of juice they were instructed to. Both the placebo-juice and bilberry/red grape juice were well tolerated. However, 4 participants in each group had temporarily mild gastrointestinal problems over the first two weeks. In the control group two participants reported that their feces had a red color, possibly due to the natural coloring agent (carmine) used in the placebo-juice. No adverse effects were observed on renal function (serum creatinine, uric acid) and the hepatic enzyme, ASAT, using a non-parametric paired test to compare pre and post levels separately in each group (data not shown).

## Discussion

The 9-week placebo-controlled double-blinded clinical trial with European blueberry and grape-juice, conducted in aged men with SMI had no effects on memory scores but decreased biomarkers of tissue damage and inflammation. To our knowledge, the present study is the first human intervention conducted, aimed at testing the combined effects of polyphenol-rich juice on memory function and relevant biomarkers.

The lack of effect on memory function and motor scores in our study contradicts the findings of Krikorian et al. who found significant effects of 12-week grape juice supplementation on memory function in a small study of only 12 subjects [29, 31]. However, these findings were observed for verbal memory which was not part of the neurophysiological measures used in the current study. The intervention juice in our study was also different, consisting of 50% bilberry and 50% grape juice as compared to the 100% grape juice used in the Krikorian study [29]. Other factors explaining the discrepancy may be a shorter treatment period, comparatively high education-level in the present sample (median 17 yrs), long-standing working careers, a healthy lifestyle and less initial memory decline problems at baseline.

However, memory performance at the initial test screening were below CANTAB-norms in particularly for ‘delayed matching to sample’ and ‘visual paired associate learning’. Therefore, a general ceiling-effect in the current study is less likely. At screening and at first testing, it was also established that the subjects had the motor speed and understanding of instructions necessary to perform the individual tests. The CANTAB tests were chosen because of their documented validity in assessing visual memory in normal and aged subjects, and being computer generated, their relative independence of experimenter effects. To prepare for testing, and to account for an eventual learning-effect between the test-time-points, an introductory adaptation session was given. However, because the participants had a low level of familiarity and practice in dealing with PC-based tests, the introductory session was perhaps insufficient to prepare for a technically effortless execution of the tests. A potential memory intervention effect could therefore be masked by the mental challenge of executing the tests. However, given the RCT design of the study, the masking effect should be equal for the two groups. Furthermore, a relatively high educational level as in the present study group may be associated with a better ability to compensate for memory decline, and therefore mask at least a moderate positive intervention effect. For comparison, in a similar age group (> 67 years) of the Norwegian population less than 5% have attained the highest degree of education (www.statisticsnorway.no). Another possible explanation to the lack of intervention effects is that visual memory might be less affected by polyphenol intervention than verbal memory. Immediate verbal recall has indeed been shown to be especially reactive to consumption of chronic flavonoid or polyphenol berry juices in adults [44]. Also, it is possible that the sample size was too small to detect a change in the selected memory scores. Finally, it is possible that the bilberry/red grape juice have no beneficial effects for memory scores. In line with our findings, Boersplug et al. did not find effects of a 16-week daily blueberry supplementation on memory scores [45]. Also, a placebo-controlled trial of 215 participants did not find any effects of a blueberry/grape extract on PAL scores but sub-group analysis revealed a significant effect in those that initially had a lower level of memory performance [32]. However, in the current study the group effects on the changes in PAL measures were not affected by the baseline levels (ANCOVA models, data not shown).

Although we do not find effects on memory scores during the 9-week intervention we find that the bilberry/red grape intake decreases the amount of several inflammatory cytokines in plasma including IL6, TNFα, EGF, Mip1β and VEGF. These results are in line with clinical trials with grape products [18, 19] and bilberry products [20, 21, 46] suggesting that these plant foods may dampen inflammation.

Chronic inflammation has been increasingly linked with several age-related diseases and suggested to play an important role in the onset and progression of AD [47]. Increased peripheral IL6 has been found to be associated with cognitive dysfunction in various pathologies [48]. IL6 and TNFα have been reported to be increased in blood of AD patients as compared to controls [49–51]. EGF has, in a panel of 18 proteins, been demonstrated to predict AD [52]. Mip1β has been shown to be stimulated by amyloid-beta (Abeta, 1–42) in human macrophages [53]. Vascular endothelial growth factor (VEGF) signals the proliferation and migration of endothelial cells in angiogenesis and has been reported to be elevated in auto-immune disease [54]. IL 10, reckoned to be anti-inflammatory, was observed to be significantly down-regulated by the juice intervention. However, IL10 has been reported at higher levels in patients with dementia compared to healthy controls [49, 50] and brain IL-10 levels are increased in neurological diseases, including AD [55]. At baseline, several cytokines (IL6, IL8, IL17, GM-CSF, TNFα, IL9, IFNγ and VEGF) correlated significantly with negative memory scores such as PAL mean trials to success and/or PAL total errors and IL12 and IL9 were inversely correlated with positive memory scores (SRM numbers correct; data not shown). Thus, there might be a potential for a health-beneficial dampening of inflammation in this population. In line with our findings a 16-week intervention study found beneficial effects on neuronal activation in MCI individuals although no effects on memory scores were evident [45].

The increase in number of monocytes in the bilberry/red grape group was also significant. The implication of this effect is unclear but indicates that the decrease in cytokine levels is due to regulation of expression levels in cytokine-producing cells rather than due to regulation of cytokine-producing cells.

The results from the current study also indicate that intake of polyphenol-rich juice may protect against tissue damage. The plasma levels of the tissue damage biomarker LDH decreased significantly in the bilberry/grape-group as compared to controls. LDH which was significantly decreased in plasma in the bilberry/red grape group compared to placebo may indicate that the intervention protects against tissue damage. As LDH, is present in several isoforms and tissues (i.e. heart, reticuloendothelial system, lungs, kidneys, liver and striated muscle) elevated levels could reflect damage to any of these organs. In line with these findings, the total creatinin kinase (CK_total_) and the heart/myocardial tissue specific, CK-MB also tended towards a differential decrease in the bilberry/red grape group compared to placebo. It is possible that the protective effects for tissue stability/renewal are related to restraining chronic inflammation, oxidative stress and the subsequent oxidative damage. Clinical trials with grape products [18, 19] and bilberry products [20, 21] suggest that these plant foods may beneficially modulate oxidative damage. Bilberries, in particular, contain high levels of antioxidants. Dietary antioxidants may possibly oppose oxidation through a direct antioxidant effect or via modulating gene expression that modulate oxidative stress defenses [40]. Also, the resveratrol component of red-grapes has been shown to reduce oxidative stress (lower malondialdehyde, nitrite levels and restoration of GSH activity) in AD rats along with improving cognitive dysfunction [56]. Furthermore, several clinical intervention trials with grape juice indicate possible protection against DNA damage and oxidative stress [19, 57].

In vivo effects of phytochemicals depend on bioavailability. Our results indicate that the phenolic compounds or their precursors are bioavailable since we observed a significant increase of several polyphenols in fasting plasma after intake of bilberry/red grape juice. Similar findings were observed in two independent Finnish 8 week intervention studies with i) 160 g/day mixed berries including bilberries [58] ii) bilberry enriched diet (puree and dried bilberries eq. 400 g fresh bilberries reporting effects on Hippuric acid [23].

Bio-available components in both the bilberries and the red grapes may therefore be candidates behind the anti-inflammatory effects measured in the current study. A recent in vitro experiment showed that LPS challenged rat cells had a decrease in oxidative stress- and inflammation biomarkers when added serum from humans that had ingested freeze-dried whole berry powder from blueberries and strawberries. The results indicates that berry metabolites, presented in blood following ingestion, may indeed be responsible for the anti-inflammatory effects of dietary berries [59]. We have also previously shown that anthocyanins isolated from bilberries and black currants inhibit nuclear factor-kappaB activation in monocytes and reduce plasma concentrations of pro-inflammatory mediators in healthy adults [21].

The mechanisms behind the anti-inflammatory properties of red grapes have mainly been reported for the phenolic compound; resveratrol. Resveratrol lead to down-regulation of the pro-inflammatory factor NF-κβ through SIRT1 activation [60]. Also, intake of bilberries reduced the development of chronic inflammation in a high-fat mouse model [61].

One theory to explain the link between inflammation and neurodegenerative diseases is that vicious circles of inflammation will lead to cumulative molecular modifications and (e.g., telomere shortening, DNA damage, epigenetic modifications, lysosomal dysregulation) and that damaging effects over time is causing the clinical manifestation of the diseases [47]. In line with this theory Wendeln et al. demonstrated that repeated LPS injection can cause epigenetic changes in mouse microglial cells for up to six months with potential cumulative and long-lasting changes [62]. It is therefore possible that a longer-time frame would be needed to establish whether the anti-inflammatory effects of the bilberry/red grape juice intake will manifest in neurological protection [63].

We cannot rule out the possibility that the pasteurization process may have affected the polyphenol content of the experimental juice and thus attribute the lack of effect on cognition. However, we have analyzed the total antioxidant content of more than 3100 foods and beverages and observed that the total content of antioxidants in general is well preserved by heating, pasteurization and other food preparation methods [64]. We have also analyzed numerous polyphenol-rich juices and observed that they all contain high amounts of total antioxidants. The reason for no effect on cognition is therefore not likely to be explained by loss of polyphenols during pasteurization of the juice. In addition, the findings that several plasma polyphenols increased after intake of the bilberry/grape juice and that there seems to be an effect to dampen inflammation, indicate that the polyphenols were both bioavailable and bioactive.

It is however possible that the baseline intake of polyphenol-rich food was already too high to be able to induce an additional health effect by the grape/bilberry intervention. The baseline dietary recordings show that both groups of the study population had a very high mean intake of fruits, vegetables (including potatoes) and berries (689 g/day and 670 g/day respectively). In addition the intake of polyphenol-rich juices was much higher than previously reported for Norwegian males at comparable age (60–79 years), which was 439 g/day [65]. Biomarkers of fruit and vegetable intake, the carotenoids, did not change more in the bilberry/red grape group compared to placebo during the study, indicating that intake of habitual plant foods remain similar between the groups. However, the decrease in polyphenols in the placebo group indicates that the intervention drink might have replaced other habitual polyphenol-rich drinks such as red wine. It is likely that polyphenol-rich drinks were replaced in similar matter in the bilberry/red grape group and that a higher increase in plasma polyphenols, and also larger health effects, might be expected if a run-in period with low intake of polyphenols i.e. restrictions on red wine was included before start of the intervention. The baseline characteristics and dietary intake recordings in total indicate that the advertisements for the study have attracted a study population that is highly health concerned. The window of opportunity to improve the polyphenol levels might therefore have been limited.

## Conclusions

Nine weeks of bilberry/red grape juice intervention in aged men with subjective memory impairment did not affect visual memory and psychomotor tempo. However, the intervention decreased levels of tissue damage- and inflammation biomarkers which indicate beneficial effects of the intervention possibly by restraining chronic inflammation, oxidative stress and the subsequent oxidative damage. In order to increase our knowledge related to bilberry/grape intake on memory function we suggest that studies with a longer timeframe should be performed.

## Supplementary Information

**Additional file 1.**

**Additional file 2.**

## Data Availability

The datasets used and/or analyzed during the current study are available from the corresponding author on reasonable request.

## Abbreviations

33HPPA: 3-(3-hydroxyphenyl)-propionic acid
3HPAA: 3-hydroxyphenylacetic acid
AD: Alzheimers disease
ASAT: Aspartate aminotransferase
BMI: Body Mass index
CANTAB: Cambridge Cognition Test Battery
CK: Creatine kinase
CL: Confidence Level
DHCA: Dihydrocaffeic acid
DMS: Delayed matching to sample
DOPAC: 3,4-dihydroxyphenylacetic acid
EDTA: Ethylenediaminetetraacetic acid
EGF: Epidermal growth factor
FFQ: Food frequency questionnaire
FGF: Fibroblast growth factor
FRAP: Ferric reducing antioxidant power
γGT: γ-glutamyl transferase
GM-CSF: Granulocyte-macrophage colony-stimulating factor
HDL: High density lipoprotein
HPLC: High performance liquid chromatography
HVA: Homovanillic acid/4-hydroxy-3-methoxyphenylacetic acid
IFN: Interferon
IL: Interleukin
IP: Immunoprotein
LDH: Lactate dehydrogenase
LDL: Low density lipoprotein
MADRS: Montgomery Asberg Depression Rating Scale
MCI: Mild cognitive impairment
MCP: Monocyte chemotactic protein
MIP: Macrophage inflammatory protein
MMSE: Mini-Mental State Examination
PA: Protocatechuic acid
PAL: Paired associates learing
PGF2: 8-epi-prostaglandin F2
PRM: Pattern recognition memory
SCI: Subjective cognitive impairment
SMI: Subjective memory impairment
SRM: Spatial recognition memory
TNF: Tumor necrosis factor
VEGF: Vascular endothelial growth factor
