# Supplementary material for: Bilberry/red grape juice decreases plasma biomarkers of inflammation and tissue damage in aged men with subjective memory impairment –a randomized clinical trial
Source: BMC Nutr. 2021 Nov 22;7:75. doi: 10.1186/s40795-021-00482-8 (PMC8607697; doi:10.1186/s40795-021-00482-8)
Supplement: Supplementary file 1 — Additional file 1. [file 40795_2021_482_MOESM1_ESM.pdf]

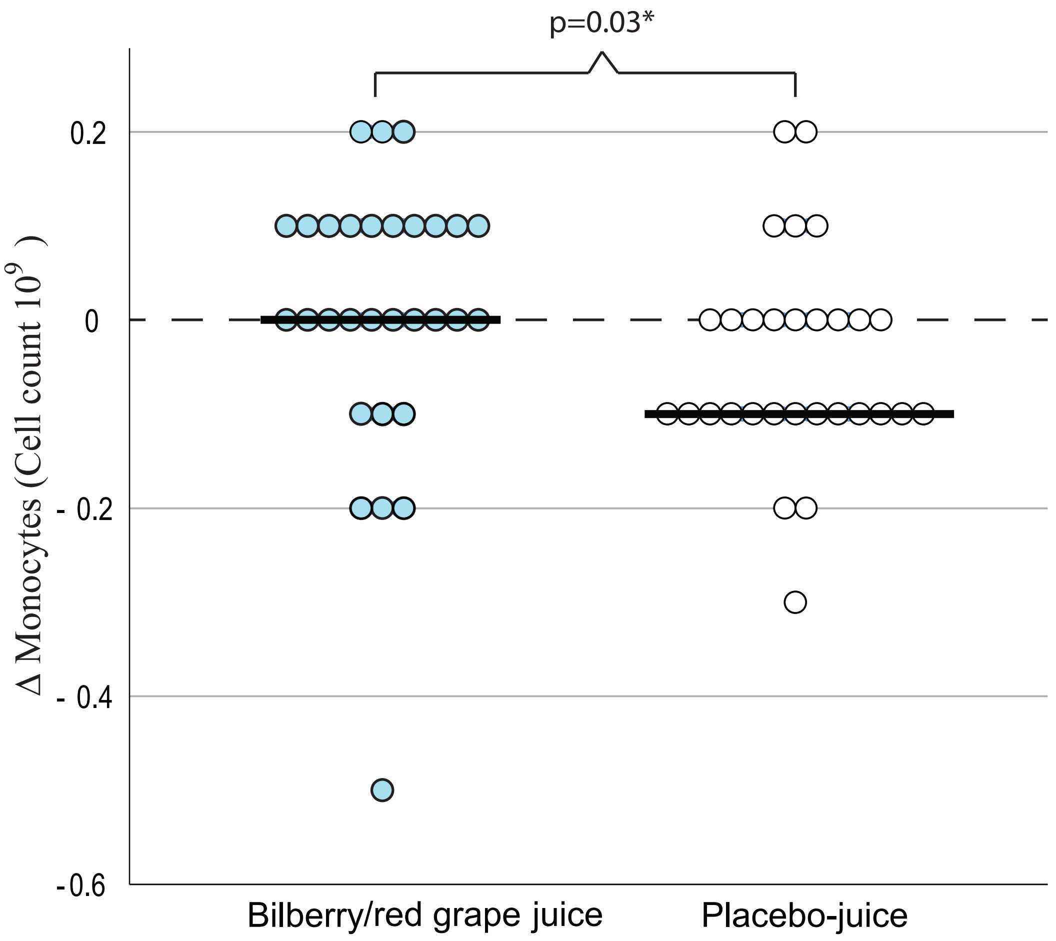

Changes in the number of monocytes in the Bilberry/red grape group (blue) and the placebo-juice group (white). P-value is obtained by non-parametric MW test.
