# Supplementary material for: Bilberry/red grape juice decreases plasma biomarkers of inflammation and tissue damage in aged men with subjective memory impairment –a randomized clinical trial
Source: BMC Nutr. 2021 Nov 22;7:75. doi: 10.1186/s40795-021-00482-8 (PMC8607697; doi:10.1186/s40795-021-00482-8)
Supplement: Supplementary file 2 — Additional file 2. [file 40795_2021_482_MOESM2_ESM.pdf]

Supplemental Figure 2

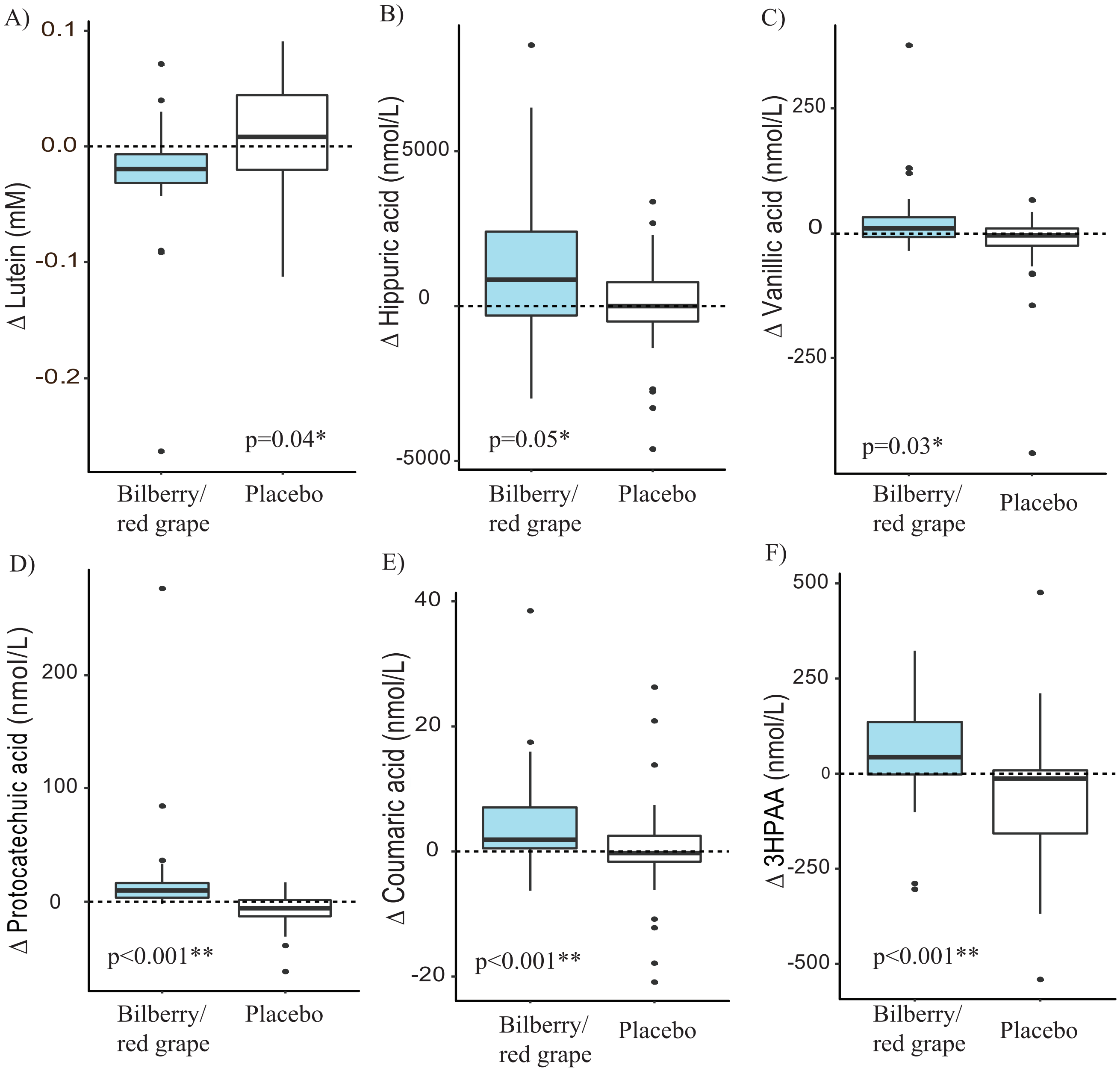

Change in lutein (A), and various polyphenols (B-F) in the bilberry/red grape juice group (blue boxes) compared to the placebo group (white boxes). P-values are obtained by non-parametric MW tests.
